# Insights into the function of ESCRT and its role in enveloped virus infection

**DOI:** 10.3389/fmicb.2023.1261651

**Published:** 2023-10-06

**Authors:** Chunxuan Wang, Yu Chen, Shunlin Hu, Xiufan Liu

**Affiliations:** ^1^Animal Infectious Disease Laboratory, College of Veterinary Medicine, Yangzhou University, Yangzhou, China; ^2^Jiangsu Co-innovation Center for Prevention and Control of Important Animal Infectious Diseases and Zoonosis, Yangzhou University, Yangzhou, China; ^3^Jiangsu Key Laboratory of Zoonosis, Yangzhou University, Yangzhou, China

**Keywords:** ESCRT, MVBS, enveloped virus, viral entry, viral budding, viral replication

## Abstract

The endosomal sorting complex required for transport (ESCRT) is an essential molecular machinery in eukaryotic cells that facilitates the invagination of endosomal membranes, leading to the formation of multivesicular bodies (MVBs). It participates in various cellular processes, including lipid bilayer remodeling, cytoplasmic separation, autophagy, membrane fission and re-modeling, plasma membrane repair, as well as the invasion, budding, and release of certain enveloped viruses. The ESCRT complex consists of five complexes, ESCRT-0 to ESCRT-III and VPS4, along with several accessory proteins. ESCRT-0 to ESCRT-II form soluble complexes that shuttle between the cytoplasm and membranes, mainly responsible for recruiting and transporting membrane proteins and viral particles, as well as recruiting ESCRT-III for membrane neck scission. ESCRT-III, a soluble monomer, directly participates in vesicle scission and release, while VPS4 hydrolyzes ATP to provide energy for ESCRT-III complex disassembly, enabling recycling. Studies have confirmed the hijacking of ESCRT complexes by enveloped viruses to facilitate their entry, replication, and budding. Recent research has focused on the interaction between various components of the ESCRT complex and different viruses. In this review, we discuss how different viruses hijack specific ESCRT regulatory proteins to impact the viral life cycle, aiming to explore commonalities in the interaction between viruses and the ESCRT system.

## 1. Structural basis of ESCRT

The Endosomal Sorting Complexes Required for Transport (ESCRT) system is a peripheral membrane protein complex composed of ESCRT-0, ESCRT-I, ESCRT-II, ESCRT-III, VPS4-VTA1, and several accessory proteins including ALIX (Johnson et al., 2018; Zhang et al., 2021). They are recruited to distinct cellular locations to perform key steps in essential processes such as protein degradation, cell division, and membrane sealing (Migliano et al., 2022). The ESCRT system was initially discovered in yeast cells (Rothman and Stevens, 1986) and subsequently found to exist in insects, mammalian cells, and even archaea. It is evolutionarily conserved in all eukaryotes and plays important roles in cytoplasmic separation (Spitzer et al., 2006), cellular autophagy (Rusten and Stenmark, 2009; Isono, 2021), other membrane fission and remodeling (Schoneberg et al., 2017), as well as replication and budding processes of certain enveloped and non-enveloped viruses (Wirblich et al., 2006; Broniarczyk et al., 2014; Ahmed et al., 2019; Rose et al., 2020), among other biological activities. Although proteins and complexes involved in the ESCRT pathway must work collaboratively, they are recruited sequentially and carry out distinct functions. Early-acting factors such as ESCRT-I and ALIX recognize and assist in remodeling the appropriate membrane, concentrating vesicular cargo, and recruiting late-acting factors (Hurley, 2010).

**Figure 1:**
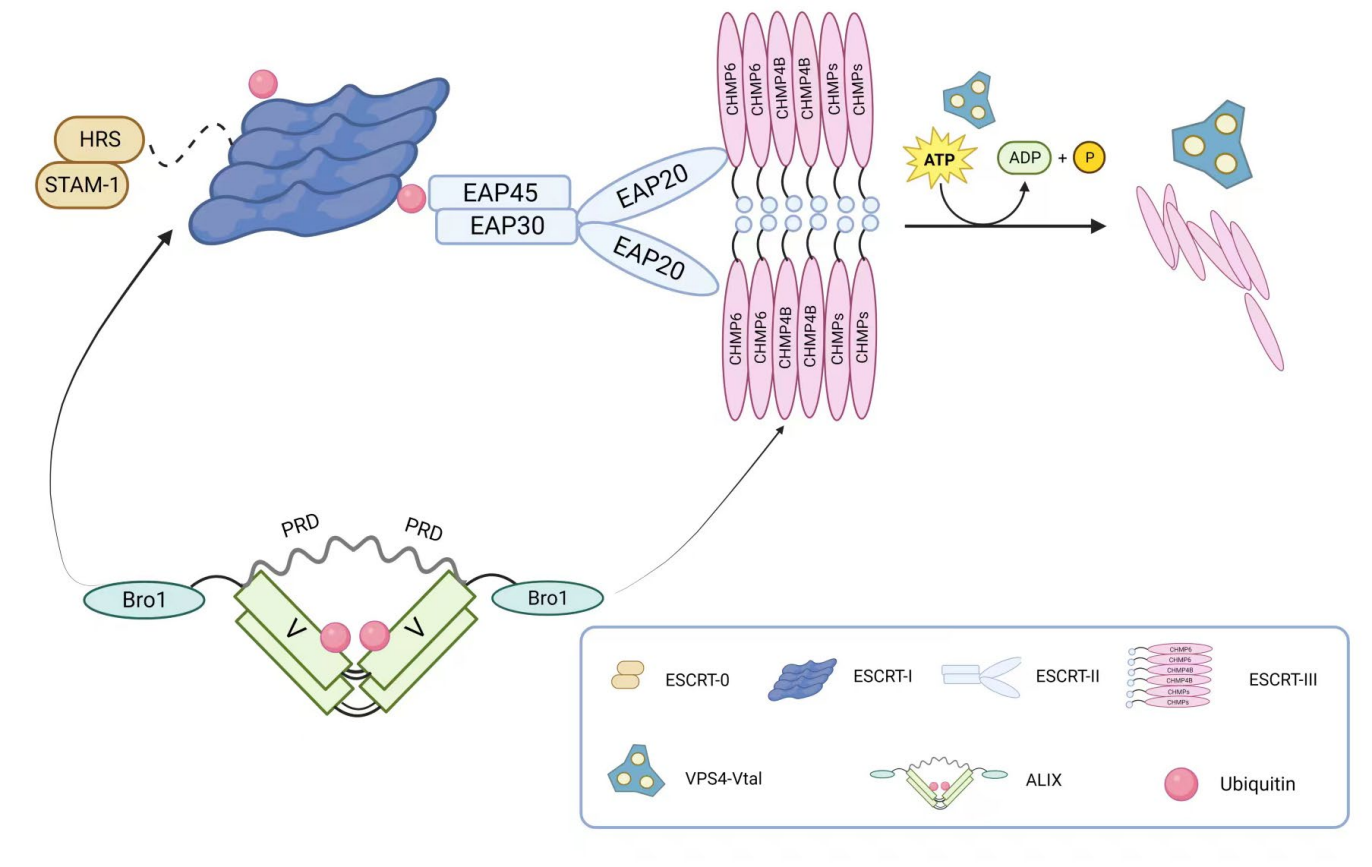
The main components of the ESCRT complex. ESCRT-0 is a dimer composed of HRS and STAM-1, primarily responsible for recognizing ubiquitinated substrates. ESCRT-I connects to ESCRT-0’s HRS through TSG101. ESCRT-II consists of one EAP45, one EAP30, and two EAP20, forming a “Y” shaped structure. The linkage between ESCRT-I and ESCRT-II is achieved through the connection of VPS28 and EAP45. ESCRT-III assembles into a tight fila-mentous structure on the cell membrane, and ESCRT-II and ESCRT-III recruit CHMP6 through EAP20. The ESCRT-associated protein ALIX is composed of the N-terminal Bro1 domain and the C-terminal proline-rich domain (PRD), located on either side of the “V” shaped central region. Through its Bro1 domain, ALIX recruits TSG101 and CHMP4B. Membrane neck constriction and ESCRT-III disassembly are catalyzed by the ATPase VPS4. The VPS4 complex hydrolyzes ATP to provide energy for thinning the bud neck and for disassembling and recycling ESCRT-III aggre-gates through the central pore.

The core structure of ESCRT is illustrated in Figure 1. The ESCRT-0 complex, a heterodimer, primarily recognizes ubiquitinated substrates. In eukaryotes, it consists of hepatocyte growth factor-regulated tyrosine kinase substrate (HRS) and signals transducing adaptor molecule (STAM1/2), whereas in yeast cells, it is composed of VPS27 and HSE1. These subunits form a composite helix through the GAT (GGA and Tom1) domain in a 1:1 ratio (Asao et al., 1997; Prag et al., 2007). As a regulatory factor in one of the essential intracellular vesicular transport pathways, ESCRT-0 recognizes, recruits, and binds to membrane proteins on the cell membrane. It then associates with ESCRT-I and ESCRT-II complexes to form vesicles for intracellular transport and degradation of substances. Therefore, ESCRT-0 plays a critical role in regulating vesicular transport pathways in the cytoplasm.

ESCRT-I, structurally forming an elongated heterotetramer, was the first discovered ESCRT structure. It consists of VPS23/TSG101, VPS28, VPS37, and MVB12/UBAP1 (Ubiquitin Associated Protein 1) in a 1:1:1:1 ratio. On one hand, the UEV domain of VPS23/TSG101 interacts with ESCRT-0 and ubiquitinated membrane proteins. ESCRT-I can bind two ubiquitin moieties on the endosome due to the association of UBAP1 and yeast MVB12 with ubiquitin. On the other hand, VPS28 interacts with the GLUE domain of ESCRT-II protein EAP45, thereby interacting with the ESCRT-II complex. Additionally, ESCRT-I and Alix can recruit ESCRT-III components, which mediate the final scission step during cell division. Moreover, ESCRT-I is involved in regulating various cellular processes such as viral infection (Morita et al., 2007) and apoptosis (Morita et al., 2007; Kaul and Chakrabarti, 2017).

As a key factor in intracellular membrane transport and degradation pathways, ESCRT-II participates in forming and dissociating intracellular vesicles in the endolysosomal system. In yeast cells, this complex consists of VPS36, VPS25, and VPS22, while in mammals, it is composed of the endosomal sorting complex required for transport-associated proteins (EAP). The Y-shaped structure of ESCRT-II is formed by EAP30, EAP45, and EA20 in a 1:1:2 ratio (Alam et al., 2006). Similar to ESCRT-I, ESCRT-II has only one ubiquitin-binding domain, which limits its ability to recruit ubiquitinated proteins. However, it contains domains that interact with ubiquitin (Slagsvold et al., 2005), ESCRT-I (Teo et al., 2006), and specific membrane components. Therefore, it does not directly participate in sorting ubiquitinated proteins but functions after cargo selection by ESCRT-0. Additionally, the SNF8 protein (VPS22) of ESCRT-II interacts with calcium transporters, thereby participating in receptor-mediated calcium signaling in various cells and tissues (Carrasquillo et al., 2012).

In contrast to early ESCRT complexes (ESCRT-0, -I, and -II) that form stable protein complexes in the cytoplasm, the ESCRT-III complex assembles only transiently on endosomes. ESCRT-III belongs to the CHMP family, soluble monomers that are widely present in eukaryotes and participate in intracellular membrane transport and degradation pathways. It also acts as a membrane remodeling machinery, providing the driving force for membrane constriction at the neck and subsequent scission (Fabrikant et al., 2009; Wollert and Hurley, 2010). The ESCRT-III complex consists of 12 proteins, including CHMP1-7 and IST1, and their recruitment and assembly are regulated by early ESCRT complexes (ESCRT-0, -I, and -II). In most cases, ESCRT-III exists as filaments in cells, assembling on membranes to form tight helical structures. It mediates the rupture and sealing of various cellular membranes, including those between the nuclear envelope and plasma membrane, and is released from the membrane in the final stage through the transient ATP-dependent reaction with other ESCRT proteins, facilitated by Vps4. Furthermore, several studies have shown that the ESCRT-III complex plays important roles in various biological processes, including cell division (Harker-Kirschneck et al., 2022; Hurtig et al., 2023), immune regulation (Li et al., 2022), and neurodegenerative diseases (Lee et al., 2007a; Han et al., 2012).

The AAA-ATPase complex Vacuolar protein sorting 4 (VPS4) consists of the type I AAA-ATPase Vps4 and its cofactor Vta1. By harnessing the energy provided by ATP hydrolysis, it disassembles the ESCRT-III complex on endosomal membranes, enabling recycling. Upon completion of ESCRT-III disassembly, the Vps4 complex dissociates into its inactive protomers. Therefore, the Vps4 complex terminates each round of MVB cargo sorting and vesicle formation (Xiao et al., 2007; Shestakova et al., 2010). Two types of VPS4 complexes, VPS4A and VPS4B, have been identified in mammals, and they play important roles in cell biology and disease, including cell division (Morita et al., 2010), membrane fusion and fission (Finken-Eigen et al., 1997; Migliano and Teis, 2018), and viral infection (Kieffer et al., 2008; Broniarczyk et al., 2017) among other biological processes. Nearly all viruses that utilize ESCRT budding require the recruitment of VPS4, indicating its critical role in viral budding (Hurley and Hanson, 2010).

ALIX protein is a multifunctional cellular protein with a structure depicted in Figure 1. It is primarily composed of three domains: Bro1, V, and PRD (proline-rich domain). Each domain mediates interactions with different cellular and protein partners (Bissig and Gruenberg, 2014). The interaction between AAA-ATPase complex Vacuolar protein sorting 4 (VPS4) and apoptosis-linked gene 2 (ALG-2)-interacting protein X (ALIX) is a potential determinant of ESCRT-I and ESCRT-II function, promoting certain ESCRT-mediated activities through direct interactions with ESCRT-I and ESCRT-III. ALIX can also associate with various effector and adaptor proteins, such as TSG101 (Strack et al., 2003; von Schwedler et al., 2003), tyrosine kinases (Schmidt et al., 2005), and CEP55 (centrosomal protein 55; Carlton et al., 2008), and these interactions are crucial for many viral budding processes. Furthermore, ALIX exhibits diverse functions, including promoting exocytosis (Fan et al., 2019), cell migration (Xie et al., 2023), and apoptosis (Strappazzon et al., 2010).

## 2. Functions and mechanisms of the ESCRT complex

**Figure 2:**
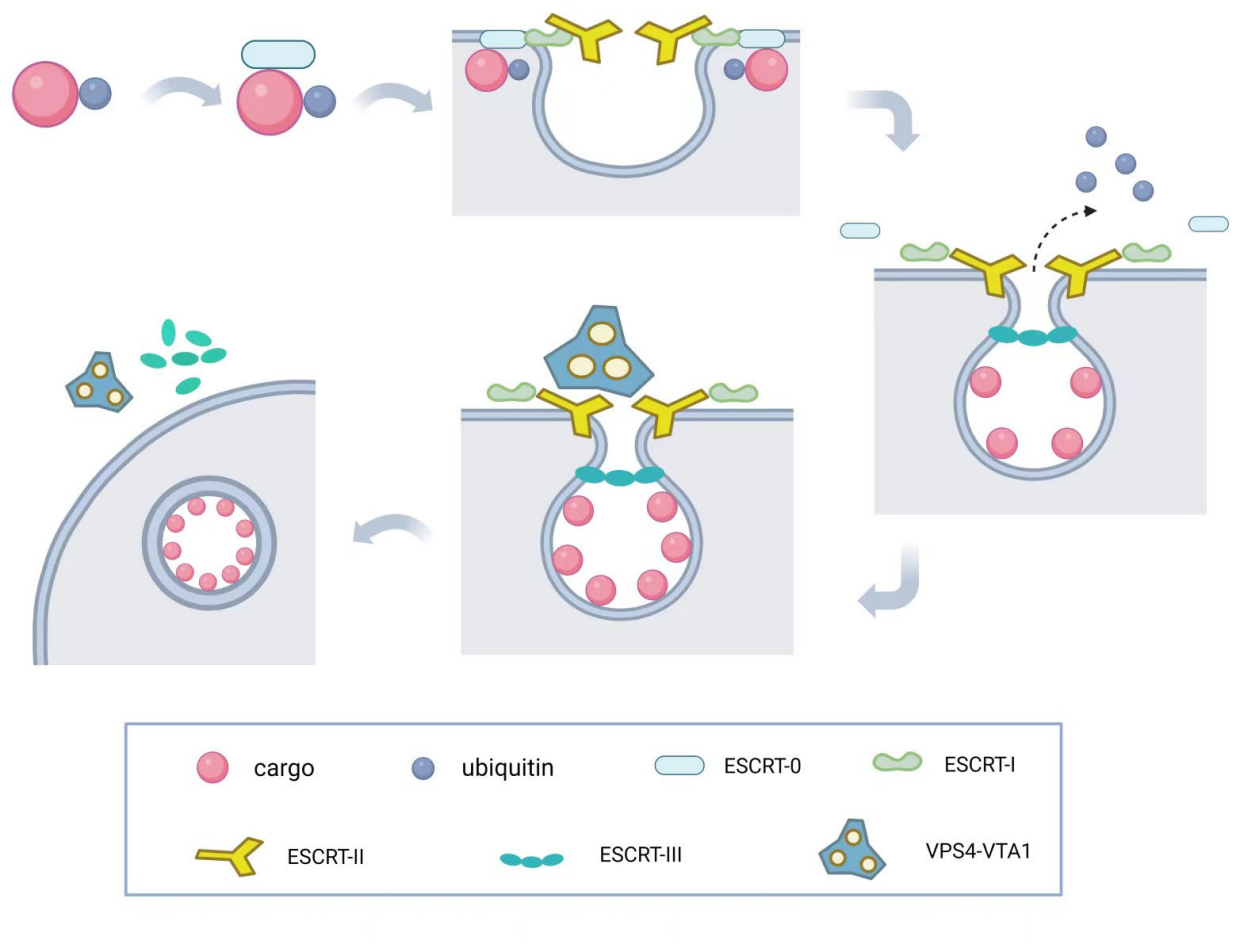
Molecular mechanism of ESCRT complex. The ESCRT-mediated budding process involves a series of coordinated steps. First, ESCRT-0 recognizes and enriches ubiquitinated substrates to recruit ESCRT-I and initiate its budding process. Then, ESCRT-I and ESCRT-II interact to induce invagination of the endosomal membrane, forming the initial bud. Subsequently, ESCRT-III cleaves the bud neck to release the cargo into the endosomal lumen, completing the budding process. Finally, the VPS4-VTA1 complex uses ATP hydrolysis to provide energy, dissociating the ESCRT-III complex from the endosomal membrane and allowing the recycling of ESCRT machinery and the endosomal membrane to occur.

The molecular mechanism underlying membrane invagination is illustrated in Figure 2. Initially, ESCRT-0 recognizes and enriches ubiquitinated substrates to recruit ESCRT-I and initiate the budding process. Ubiquitination serves as a critical entry point for the ESCRT pathway and plays a crucial role in retroviral budding (Strack et al., 2000). Ubiquitinated cargo molecules are sorted into MVBs, where proteins such as TSG101, UBAP1, EAP45, and ALIX contain ubiquitin-binding sites. ESCRT-I and ESCRT-II then interact with each other to induce inward membrane invagination and form initial buds. Subsequently, ESCRT-III cuts the neck of the buds, releasing small vesicles into the lumen of the endosome, completing the budding process. Finally, the VPS4-VTA1 complex, powered by ATP hydrolysis, dissociates the ESCRT-III complexes from the endosomal membrane, separating the ESCRT structures and allowing for recycling. Once MVBs fuse with lysosomes, the intraluminal vesicles (ILVs) are degraded within the lysosomal lumen. Therefore, the ESCRT system plays a crucial role in sorting substances and downregulating surface receptors, while also participating in the precise regulation of cell signaling (Taelman et al., 2010; Sigismund et al., 2012). During this process, upstream ESCRT mechanisms or other recruiting factors, such as viral Gag proteins, initiate membrane deformation and allow downstream ESCRT machinery to assemble in the absence of pre-existing membrane necks or pores. ESCRT-III components are recruited by ESCRT-I/-II and/or Bro domain proteins (Christ et al., 2016) and preferentially aggregate on curved membranes (Lee et al., 2015; de Franceschi et al., 2019; Bertin et al., 2020; Nepal et al., 2020). The following sections will describe several important functions of the ESCRT complex.

### 2.1. ESCRT and autophagy

Degradation and recycling of intracellular substances are fundamental mechanisms for maintaining cellular homeostasis and responding to adverse external conditions. Most cells employ several distinct degradation systems: the ubiquitin-proteasome degradation system, endocytosis, autophagy, and lysosomal degradation system (Lefebvre et al., 2018). Among these, autophagy is a conserved cellular mechanism in all eukaryotes, through which organelles, proteins, and invading pathogens can be sequestered and degraded within vesicles (Marshall and Vierstra, 2018). Within cells, various types of autophagic pathways coexist, relying on different combinations of cellular and molecular mechanisms, but ultimately delivering cargo for degradation in lysosomes (Rusten and Stenmark, 2009). The ESCRT machinery is capable of participating in different types of autophagic processes to degrade cytoplasmic components, such as macroautophagy and microautophagy. The distinction between the two lies in the fact that, in macroautophagy, autophagic substrates are transported to vesicles called autophagosomes, while in microautophagy, substrates are directly engulfed by invaginations of the vacuolar membrane.

The formation of autophagosomes requires the induction, transport, and fusion of membrane-bound organelles. Previous studies have shown that disruption of the ESCRT machinery in mammals leads to autophagosome accumulation, indicating the important role of ESCRT in regulating autophagy (Filimonenko et al., 2007; Lee et al., 2007a). The relationship between ESCRT and autophagy is linked through shared molecular mechanisms involved in autophagosome formation and fusion. The ESCRT machinery rescues mildly damaged lysosomes and interacts with autophagy-related proteins (ATGs) to form a bridge between endocytic and macroautophagy processes, facilitating the formation of the endo-lysosomal system and cell death. ESCRT components have been proposed as potential regulators of autophagosome membrane closure (Hurley, 2015; Knorr et al., 2015). Proper autophagosome membrane closure requires ESCRT-III component CHMP2A and AAA-ATPase vacuolar protein sorting-associated protein 4 (VPS4) activity (Takahashi et al., 2018). Upon maturation, autophagosomes fuse with late endosomes/lysosomes for degradation, and this process involves ESCRT proteins such as ALIX (Petiot and Sadoul, 2009) and VPS4. Additionally, certain components of the ESCRT complex participate in regulating different steps of the autophagic pathway. For instance, the ESCRT-III protein SNF7 plays a role in autophagosome formation and membrane sealing (Schaefer et al., 2020), while VPS4 promotes the fusion of autophagosomes with late endosomes, facilitating the progression of autophagy (Teis et al., 2010; Tang et al., 2015). Therefore, the ESCRT complex plays a crucial role in the autophagic pathway and is closely associated with the mechanisms of autophagy.

### 2.2. ESCRT-dependent membrane remodeling and fission

ESCRT is an evolutionarily conserved membrane remodeling complex with the unique ability to catalyze membrane constriction within necks. Among them, ESCRT-III, in collaboration with the AAA ATPase VPS4, plays a central role in the main membrane remodeling and scission functions of the ESCRT machinery (Stoten and Carlton, 2018). ESCRT-III exerts its membrane fission catalytic ability on almost all cellular membranes. It catalyzes the formation of ILVs within endosomal membranes, drives membrane scission from the cell surface, facilitates nuclear envelope reformation, and mediates autophagosome closure (Schoneberg et al., 2017). It is recruited to various distinct sites of action through interactions with multiple adaptor proteins. VPS4, on the other hand, is recruited to the membrane to facilitate the disassembly of ESCRT-III components (Yang et al., 2015). Cells utilize this system for reverse membrane fission during processes such as protein degradation, cell division, and retrovirus release. Retroviruses recruit ESCRT-III to bud sites to ensure cell exit. In this process, the ESCRT complex, through interaction with viral particle proteins, forms a protein membrane fission machinery that promotes viral particle release and aids in the evolution of the L domain (Scheffer et al., 2014).

Not all organisms require the complete ESCRT complex, but ESCRT-III and VPS4 appear to be indispensable, with CHMP2, CHMP3, CHMP4, and VPS4 considered essential components in membrane remodeling. However, even in the presence of ESCRT-III proteins, membrane fission is halted when AAA ATPase VPS4 is absent. Furthermore, ESCRT demonstrates critical activity in many other cellular membrane remodeling events, such as enveloped virus budding, cytokinesis abscission, exosome biogenesis (Larios et al., 2020), plasma membrane repair, immune synapse formation, and nuclear membrane homeostasis (Christ et al., 2017).

### 2.3. ESCRT and endosomal sorting

The ESCRT machinery was initially discovered through genetic studies in yeast, where mutations led to improper sorting of proteins into vesicles, functionally equivalent to lysosomes (Alfred and Vaccari, 2016). These mutant strains, known as “E-vps mutants,” exhibited enlarged pre-vacuolar endosome-like compartments containing undegraded proteins (Haguenauer-Tsapis, 2012). Subsequently, it was found that most class E VPS genes act sequentially, concentrating transport cargoes and incorporating them into late endosomes (also known as multivesicular bodies or MVBs), which eventually fuse with lysosomes for degradation (Katzmann et al., 2001). During the sorting process, various complexes of ESCRT are recruited from the cytoplasm to the inner membrane of endosomes through specific subunit interactions. These complexes facilitate the sequestration of intracellular waste, proteins, and other molecules into MVBs through the formation of endosomes. Subsequently, certain MVBs can fuse with lysosomes, directing the enclosed material into lysosomes for degradation.

ESCRT complexes play crucial roles in this process, with the ubiquitination of cargoes providing a critical signal for initial cargo binding by ESCRT-0 (Urbe, 2005). The ESCRT-0 subunits HRS and STAM, ESCRT-I subunit TSG101, and ESCRT-II subunit Vps36 all contain ubiquitin-binding domains that interact with ubiquitinated cargoes. Specifically, ESCRT-0, ESCRT-I, and ESCRT-II subunits facilitate the aggregation and recognition of membrane receptors by interacting with specific signaling molecules on the membrane. Subsequently, ESCRT-III and VPS4-VTA1 subunits act on the outer membrane of the MVB, forming a cone-shaped structure that ultimately drives MVB formation and separation of internal vesicles (Huotari and Helenius, 2011). With the assistance of ESCRT complexes, the VPS4 complex dissociates ESCRT structures from the limiting membrane, and after fusion of the MVB with lysosomes, ILVs are degraded within the lysosomal lumen. Additionally, several accessory proteins participate in the regulation of this process, such as ALIX and VPS27, which interact with different subunits of the ESCRT complex, thereby modulating endosomal sorting (Bilodeau et al., 2003; Murrow et al., 2015). Thus, the ESCRT pathway also plays a critical role in the sorting and downregulation of cell surface receptors, and it is involved in fine-tuning cell signaling pathways (Raiborg and Stenmark, 2009).

### 2.4. ESCRT and cell division: key players in membrane dynamics

ESCRT was initially discovered as a regulator of intracellular cargo sorting (Katzmann et al., 2001; Babst et al., 2002). Recent studies have shown that ESCRT is also involved in a series of critical events during cell division, such as gametogenesis, chromosome segregation, and cytokinesis (Mathieu et al., 2022). The final stage of cell division is abscission, which involves the severing of the narrow membrane bridge between the two daughter cells (Steigemann and Gerlich, 2009). ESCRT is essential for cell division, and its role in cytokinesis can be traced back to ancient bacteria, suggesting that the ancestral function of these proteins was to promote membrane remodeling for division and was selected throughout evolution to perform various other cellular biological functions. In mammalian cell lines, the ESCRT-III complex mediates abscission, with the bridging protein Cep55 considered a key regulatory factor in abscission. This is due to its recruitment of ESCRT-III to the site of abscission, termed the midbody (MB; Tedeschi et al., 2020). Cep55 recruits ESCRT-III subcomplexes, subsequently facilitating membrane constriction and rupture of the intercellular bridge (Carlton and Martin-Serrano, 2007; Mierzwa and Gerlich, 2014; Addi et al., 2018). During this process, ESCRT-associated components are sequentially recruited to the membrane to mediate the scission event (Wollert et al., 2009b) and exhibit membrane-severing activity *in vitro* (Wollert et al., 2009a). The charged multivesicular body protein 1B (CHMP1B) of ESCRT-III interacts with the microtubule-severing enzyme spastin, which is crucial for cell division (Yang et al., 2009). The ESCRT-I subunit tumor susceptibility gene 101 (TSG101) and the ESCRT-III subunit charged multivesicular body protein 4B (CHMP4B) are sequentially recruited to the central region of the intercellular bridge, forming a series of cortical rings. In the late stage of cell division, CHMP4B is acutely recruited to the constricted site where abscission occurs, and VPS4 follows CHMP4B to that location, triggering immediate cell separation (Carlton and Martin-Serrano, 2007). ESCRT-III and VPS4 are spatially and temporally associated with the abscission event, indicating their direct involvement in membrane scission during cytokinesis.

During cell division, ESCRT interacts with structures such as the centrosome, chromosomes, and the cytoskeletal framework, participating in the regulation of cytokinesis, chromosome segregation, and recombination, as well as gametogenesis and embryonic development. Moreover, ESCRT may be closely associated with cell fate determination during cell division, including cell fate transitions, stem cell differentiation, and tumor development (Valentine et al., 2014; Wilson et al., 2020). In conclusion, the role of ESCRT in cell division is gradually being unraveled, and it holds significant implications for a comprehensive understanding of the mechanisms underlying cell division regulation and related diseases.

### 2.5. ESCRT and cell apoptosis

Cell apoptosis is a programmed cell death and the most extensively studied form of regulated cell death. It is characterized by sequential activation of the cysteine-aspartic protease caspases (Green, 2019). This process involves chromatin fragmentation and marginalization, as well as the generation and membrane budding of apoptotic bodies (Elmore, 2007). During cell apoptosis, cells release various cell factors and vesicles containing cellular components and nuclear fragments referred to as apoptotic bodies. The ESCRT complex can participate in the formation and clearance of apoptotic bodies. Specifically, the ESCRT-0 and ESCRT-III complexes interact with vesicle-associated proteins on the cell membrane, inducing membrane curvature and eventually leading to vesicle formation and release. Subsequently, the ESCRT-III complex assembles within the cell, forming a contractile structure that can engulf and release the apoptotic bodies to the extracellular space. Under normal circumstances, the binding of TSG101 with ALIX prevents cell apoptosis, but disruption of this process may occur due to dysregulation of cellular solute Ca2+ and subsequent upregulation of ALG-2 (Yang et al., 2022). Furthermore, the ESCRT-III complex can facilitate the release of mitochondrial content through extracellular vesicles, thereby participating in the process of apoptosis. ESCRT-III plays a crucial role in repairing damaged membranes involved in various types of regulated cell death, such as necroptosis, pyroptosis, and ferroptosis. Inhibition of the ESCRT-III mechanism through genetic depletion of its core components increases susceptibility to cell death induced by anticancer drugs, indicating that ESCRT-III could be a potential target to overcome drug resistance during cancer treatment. Therefore, the ESCRT complex plays a significant role in regulating the release and clearance of cellular components during cell apoptosis, maintaining normal physiological processes within the organism (Yang et al., 2022).

Furthermore, cells undergoing necroptosis do not always lead to cell death. The known repair mechanisms of ESCRT involve vesiculation or shedding of damaged membranes and play a critical balancing role in sorting and downregulating activated cell surface receptors (Raiborg and Stenmark, 2009) and repairing damaged membranes to maintain membrane integrity (Christ et al., 2017). This delays the cell death pathway, including cell apoptosis, necroptosis, pyroptosis, and ferroptosis (Jimenez et al., 2014; Gong et al., 2017; Ruhl et al., 2018). Cell apoptosis, necroptosis, and pyroptosis are all passive “suicidal” forms of molecular processes that result in the formation of several nanometer-sized membrane pores and catastrophic cell rupture (Espiritu, 2021). In summary, ESCRT provides time for dying cells, and ESCRT-dependent membrane repair negatively regulates cell death: ESCRT-I and -III are involved in cell apoptosis, while ESCRT-III mediates necroptosis, pyroptosis, and ferroptosis (Table 1).

**Table 1:** Interactions between different types of enveloped viruses and various ESCRT components.

Virus category	Virus name	ESCRT components	References
	NDV	ESCRT-III	Li et al. (2013) and Pei et al. (2023)
	SeV	ALIX	Kurotani et al. (1998)
	HPIV1	ALIX	Boonyaratanakornkit et al. (2013)
	NIV	ESCRT-I	Park et al. (2016)
	HIV-1	ESCRT-I, -III, VPS4, ALIX	Meng and Lever (2013), Sette et al. (2013), and Johnson et al. (2019)
	HCV	ESCRT-0, -III, VPS4	Ariumi et al. (2011) and Barouch-Bentov et al. (2016)
Enveloped RNA viruses	PRRSV	ESCRT-I	Zhang et al. (2022)
	EBOV	ESCRT-I, ALIX	Han et al. (2015)
	MARV	ESCRT-I	Dolnik et al. (2010, 2014)
	LASV、LCMV	ESCRT-0, -I, ALIX	Pasqual et al. (2011) and Shtanko et al. (2011)
	RABV	ESCRT-I, -III, ALIX	Finke and Conzelmann (2005) and Itakura et al. (2023)
	IHNV	ESCRT-I, VPS4, ALIX	Chen et al. (2019)
	CSFV	ESCRT-0, -I, -II, -III, ALIX	Liu et al. (2021, 2022)
Enveloped DNA viruses	HBV	ESCRT-0, -I, -II, -III, VPS4	Stieler and Prange (2014), Chou et al. (2015), and Zheng et al. (2023)
	HSV-1	ESCRT-III	Crump (2018) and Barnes and Wilson (2020)
	AcMNPV	ESCRT-I, -III, VPS4	Li and Blissard (2012) and Yue et al. (2018)

## 3. ESCRT and enveloped viruses

The ESCRT machinery is not only essential for cellular processes such as membrane repair but is also hijacked by certain viruses during different stages of replication, particularly during the budding phase. The interaction between certain ESCRT components and viral proteins is primarily mediated by short tetrapeptide motifs known as Late assembly (L) domains. These motifs mediate the recruitment and interaction of class E proteins to facilitate virus budding, envelopment of retroviruses (e.g., HIV), and other RNA viruses (e.g., filoviruses, orthomyxoviruses, and paramyxoviruses), redirecting cellular ESCRT proteins to the cytoplasmic membrane for viral particle release and egress from infected cells.

Many enveloped viruses utilize the ESCRT pathway to catalyze membrane scission, leading to the release or budding of viral particles (Votteler and Sundquist, 2013). This process is dependent on specific late-domain structures of the respective viruses. All viruses that bud in an ESCRT-dependent manner require the membrane-remodeling functions provided by ESCRT-III and VPS4, but their requirements for upstream factors may vary. For instance, HIV-1 recruits the ESCRT pathway through specific motifs on the viral Gag protein (Morita and Sundquist, 2004), Marburg virus and Ebola virus VP40 interact with ESCRT-associated proteins such as Alix and TSG101 through their L domains to facilitate budding (Timmins et al., 2003; Urata et al., 2007). Enveloped virus infection begins with the attachment of the virus to the host cell’s plasma membrane, followed by viral entry, replication, and expression within the host cell. Finally, mature virus particles are released from the host cell, initiating a new infectious cycle. Studies have revealed the hijacking of the ESCRT system by HIV for its budding, and subsequent research has demonstrated the hijacking of the ESCRT system by various viruses, mediating different steps of the virus life cycle in different cells, thereby facilitating virus proliferation, budding, and dissemination. The ESCRT system has become an integral component of enveloped virus infections (Votteler and Sundquist, 2013; Meng and Lever, 2021). In the following, we summarize the reported interactions between various types of enveloped viruses and different ESCRT components, aiming to enhance our understanding of the interplay between ESCRT complexes and viral infections. Additionally, we aim to provide new research directions and contribute to the development of therapeutic strategies for future studies.

### 3.1. ESCRT pathway and enveloped RNA viruses

Some envelope RNA viruses utilize the ESCRT pathway to facilitate budding and release from host cells. By interacting with ESCRT proteins, these viruses can redirect intracellular proteins to the cell membrane, promoting viral particle budding and separation. The involvement of ESCRT plays a critical regulatory role in the infection, replication, and spread of enveloped RNA viruses.

#### 3.1.1. The role of ESCRT machinery in paramyxovirus budding

Avian paramyxoviruses, including Newcastle disease virus (NDV), are single-stranded RNA viruses that can cause highly contagious diseases in various avian species. The M protein of paramyxoviruses has been identified as a nucleocapsid-associated protein that plays a crucial role in the virus life cycle (Pentecost et al., 2015). During early infection, the M protein typically enters the cell nucleus through its nuclear localization signal and transiently accumulates in the nucleolus. Previous studies have shown that this M protein aggregation, observed in respiratory syncytial virus (RSV), NDV, and measles virus (MeV), can affect the transcription, synthesis, and post-translational modifications of host proteins (Yu et al., 2016). The M protein contains conserved peptides known as the L domain, which can utilize the ESCRT complex to facilitate paramyxovirus release. However, single amino acid mutations in this domain can result in severe budding defects in paramyxoviruses (Duan et al., 2014). A novel late domain sequence, FPIV, was found in the parainfluenza virus 5 (SV5) matrix protein (Schmitt et al., 2005). Studies have shown that mutation of the FPIV-like late domain sequences dramatically impairs the budding process of SV5 (Schmitt et al., 2005) and MuV (Li et al., 2009). A similar sequence motif, FPIV, is present in the NDV M protein, suggesting that this motif could play an essential role in the replication and budding process of NDV. Previous research has demonstrated the critical significance of the FPIV motif within the NDV M protein, with residues F and P playing a pivotal role in NDV replication and budding. Furthermore, the FPIV motif’s recruitment of cellular multivesicular body (MVB) machinery is implicated in NDV budding (Duan et al., 2014). As a core component of the ESCRT-III complex, CHMP4B has been identified as a key factor linking the FPIV L domain and the ESCRT machinery. Previous studies by Li et al. (2013) confirmed that the binding between NDV M protein and CHMP4 promotes virus replication. Similarly, the study by Pei et al. (Pei et al., 2023) demonstrated that the ESCRT-III complex CHMP4s directly interact with NDV M protein through the FPIV L domain, independently of TSG101, ESCRT-II, or ALIX as assembly factors. In summary, critical amino acid mutations in the FPIV L domain prevent effective interaction between NDV M protein and CHMP4B, resulting in the stalling of viral sub-particles during membrane budding and leading to defects in NDV release and growth. However, further research is needed to elucidate the detailed mechanisms underlying the interaction between NDV and the ESCRT system, as well as whether other ESCRT components are utilized to facilitate virus propagation.

Sendai virus (SeV), belonging to the Paramyxoviridae family and Respirovirus genus, is a pneumotropic virus in rodents and one of the most extensively studied members of the Respirovirus genus (Henrickson, 2003). Its structure contains a multifunctional accessory protein called the C protein, which enhances the virulence of the virus by stimulating viral replication (Oda et al., 2021). It is considered that the C proteins are not involved in virus assembly, since they are essentially nonstructural components that are abundantly expressed in infected cells but are also present in trace amounts in mature virions (Sakaguchi et al., 2005). The C proteins are categorized as nonessential accessory proteins but contribute greatly to virus replication *in vitro* and are indispensable for the *in vivo* multiplication and pathogenesis of the infection they cause (Kurotani et al., 1998). It is thought that the C protein is involved in virus budding because of the low efficiency of release of progeny virions from C-knockout virus-infected cells and because of the requirement of the C protein for efficient release of virus-like particles. In addition, the C-protein knockout [4C(−)] virus produced largely noninfectious progeny virions with a highly anomalous morphology. Excessive accumulation of viral proteins and genomic RNAs was also observed in 4C(−) virus-infected cells. These results suggest that the late step of budding is abrogated in the absence of the C protein (Hasan et al., 2000). Oda et al. conducted virus infection experiments using SeV generated with the Y3: Bro1 complex crystal structure and analyzed the impact of the interaction between SeV’s C protein and Alix, a component of the ESCRT complex, on SeV budding. Their study revealed that a decrease in the affinity between the C protein and Alix significantly reduced the infectivity of the virus, indicating that the binding of the C protein to Alix enhances SeV budding (Kurotani et al., 1998). Furthermore, some studies have suggested that Alix also regulates cellular immune responses and inflammatory reactions, which may be associated with the immune response and inflammation triggered by SeV infection, although the specific mechanisms remain unclear (Yan et al., 2014; Chen and Yan, 2015; Chen et al., 2021). While the role of the ESCRT system in other viruses has been extensively studied, there is currently a lack of comprehensive research on the interaction between the Sendai virus and the ESCRT system.

Human parainfluenza virus type 1 (HPIV1) is a common respiratory pathogen and a single-stranded, negative-sense, non-segmented RNA virus belonging to the Paramyxoviridae family. The viral genome consists of 15,600 nucleotides and encodes six proteins from six genes. Among them, the C protein serves as an important virulence factor in HPIV1. It can inhibit apoptosis, influence host gene transcription, and regulate the production and signaling of type I interferon (Van Cleve et al., 2006; Bartlett et al., 2008; Boonyaratanakornkit et al., 2009). The C protein of HPIV1 interacts with the Bro1 domain of Alix at a specific site, which is also essential for the interaction between Alix and CHMP4B. The C protein is ubiquitinated and subject to proteasome-mediated degradation, but its interaction with Alix’s Bro1 domain protects it from degradation. Studies have shown that SeV shares a high degree of homology with the C protein of HPIV1 (Andrejeva et al., 2004). However, the major difference between them is that SeV also expresses another accessory protein called the V protein, which has inhibitory effects on the host’s innate antiviral response. In contrast, HPIV1 does not express the V protein, and the C protein is the only known viral antagonist of innate immune responses. Boonyaratanakornkit et al. (2013) proposed that during HPIV1 infection, Alix binds to the C protein and recruits it to the cytoplasmic surface of late endosomes. Subsequently, CHMP4 may recruit the Alix-C core shell to a common site on the cytoplasmic surface of late endosomes, which serves as the source for virus assembly and budding.

Nipah virus (NIV) is a deadly zoonotic virus belonging to the Paramyxoviridae family, capable of causing severe respiratory and neurological diseases in humans and animals. The M protein of NIV is considered to play a central role in virus assembly and budding (Patch et al., 2007; Harrison et al., 2010). When expressed alone, the M protein is released from the cells as virus-like particles, and its budding is independent of the ESCRT pathway (Patch et al., 2008). The C protein of NIV acts as an accessory factor to counteract innate immunity, enhancing the budding of the M protein through the ESCRT pathway. Research by Park et al. (Park et al., 2016) indicates that NIV-C directly interacts with the ESCRT factor TSG101, which is essential for NIV-C to enhance budding and efficiently release infectious Nipah virus. Further studies are being conducted to gain a better understanding of the interaction mechanisms between NIV and the ESCRT system.

#### 3.1.2. The ESCRT machinery is essential for the proper assembly and budding of retroviral particles

Human Immunodeficiency Virus (HIV) is a single-stranded RNA virus belonging to the Retroviridae family. It primarily infects human immune cells, leading to immune system impairment. HIV-1 Gag is synthesized in the cytoplasm and subsequently directed to the plasma membrane, where virus assembly takes place (Lippincott-Schwartz et al., 2017). The formation of HIV viral particles occurs through the multimerization of the Gag precursor on the plasma membrane, and the budding of the virus is mediated by the interaction between Gag and various ESCRT (Endosomal Sorting Complex Required for Transport) proteins. Early components of the cellular ESCRT system, ESCRT-I (TSG101/VPS23) and ALIX mediate HIV-1 budding, both of which interact with the P6 domain of Gag. Within this domain, there are PS/TAP and YPXnL motifs that simultaneously promote virus budding, with the PS/TAP motif binding to ESCRT-I’s TSG101 and the YPXnL motif binding to ESCRT-III-associated ALIX protein. Both pathways require downstream ESCRT-III and VPS4 ATPase (Zavitz et al., 2008; Meng and Lever, 2013; Sette et al., 2013). Additionally, this domain can interact with ESCRT-II’s EAP20, directing HIV particles to the cell membrane and facilitating particle release (Pincetic et al., 2008; Medina et al., 2011; Ghoujal et al., 2012). However, Langelier et al. suggested that HIV-1 release is ESCRT-II-independent since siRNA depletion of ESCRT-II subunit EAP20 did not significantly impact virus release and infectivity. Nevertheless, they did report detrimental effects on virus production at later time points following ESCRT-II knockdown (Langelier et al., 2006). Furthermore, the Gag protein interacts with multiple members of the ESCRT-III complex, including CHMP2, CHMP3, CHMP4, and CHMP6, leading to the formation of membrane helical structures within the cell and aiding in virus particle formation and release (Baumgaertel et al., 2011; Cashikar et al., 2014; Johnson et al., 2019).

Following this, Janvier et al. (2011) investigated the additional role of the ESCRT machinery in HIV-1 release. Their research demonstrated that BST-2/tetherin, acting as a restriction factor, hinders HIV release by tethering mature virus particles to the plasma membrane. As a crucial component of ESCRT-0, HRS facilitates Vpu protein-induced downregulation and degradation of BST-2, thereby aiding in efficient HIV release. Physiologically, HRS is believed to initiate ESCRT-mediated multivesicular body (MVB) formation, with the PSAP motif in HRS recruiting the ESCRT machinery by interacting with ESCRT-I component TSG101 (Bache et al., 2003). The assembly and efficient release of HIV-1 requires highly coordinated interactions between virus-encoded proteins and cellular key components. In summary, we propose that ESCRT-0 and ESCRT-II may be dispensable for HIV budding, while Alix is indispensable. Among the ESCRT-III subunits, only CHMP4 and CHMP2 appear to be essential. Loss of ESCRT function inhibits virus release, resulting in accumulated arrested buds on the surface of infected cells.

#### 3.1.3. Interactions between HCV and the ESCRT system promote viral envelopment through ubiquitination

The Flaviviridae family comprises enveloped, single-stranded positive-sense RNA viruses, including the Hepatitis C virus (HCV), which is the causative agent of chronic hepatitis. It encodes a large polyprotein precursor of approximately 3,000 amino acid residues, which is processed by viral proteases and cellular signalizes to generate three structural proteins (core, E1, and E2) and seven non-structural proteins (p7, NS2, NS3, NS4A, NS4B, NS5A, and NS5B; Scheel and Rice, 2013). The non-structural proteins NS3, NS4A, NS4B, NS5A, and NS5B form the viral replication machinery, while p7 and NS2 are crucial for the production of infectious viruses (Jones et al., 2007; Jirasko et al., 2010). NS5A is a membrane-associated RNA-binding phosphoprotein that also participates in the assembly and maturation of infectious HCV particles (Appel et al., 2008; Tellinghuisen and Foss, 2008).

Enveloped viruses typically hijack the ESCRT system through late-domain motifs or ubiquitination to bud from the plasma membrane. However, some viruses, such as HCV, lack identifiable late-domain motifs despite lacking late structural domains, suggesting that their association with ESCRT proteins may have diverse and complex mechanisms. On one hand, the K2 ubiquitination of NS63 lysine residues mediates its interaction with the HRS ubiquitin-interacting motif (UIM) and its binding to HCV assembly, thus compensating for the apparent deficiency of the late domain. On the other hand, HCV proteins involved in virus assembly, including the core and NS2, are ubiquitinated, and the recognition of ubiquitinated NS2 and potentially other viral proteins by HRS UIM might play a role in ESCRT recruitment and HCV assembly, providing a potential mechanism by which HCV proteins could be recruited to ESCRT complexes (Shirakura et al., 2007; Welbourn et al., 2009; Barouch-Bentov et al., 2016). Subsequently, Ariumi et al. (2011) demonstrated the binding of the HCV core to CHMP4B, indicating that the core possesses a novel motif required for HCV production. According to reports by Blanchard et al., the aspartic acid at position 111 of the core protein is crucial for virus assembly. Subsequently, they introduced mutations in the core protein sequence of the Dj clone by incorporating the L domain sequence. Specifically, they changed the aspartic acid at position 111 to proline-alanine (PTDP to PTAP) through site-directed mutagenesis. The results indicated that this amino acid mutation at this position impedes the formation of HCV core particles (Blanchard et al., 2003).

HRS serves as a critical factor in HCV envelopment, while ESCRT-III and VPS4/VTA1 factors are hijacked by the HCV core and NS5A, respectively, to mediate viral assembly. The essential role of VPS4 and the ESCRT-III complex in HCV production has been demonstrated by Corless et al. (Corless et al., 2010). Deng et al. proposed that the Hepatitis C Virus (HCV) induces the Reactive Oxygen Species (ROS)/JNK (c-Jun N-terminal kinase)/Itch (HECT-type E3 ubiquitin ligase) signaling pathway, which enhances the polyubiquitination of VPS4A. This leads to an augmented interaction between VPS4A and CHMP1B, thereby promoting VPS4A ATPase activity and subsequently facilitating the release of HCV particles (Deng et al., 2022). Furthermore, integrated approaches combining proteomics, RNA interference, viral genetics, and biophysics have validated the recruitment of ESCRT components by HCV proteins to facilitate viral envelopment (Ariumi et al., 2011; Barouch-Bentov et al., 2016). ESCRT-0 component HRS has been identified as crucial for the release of HCV *via* exosome secretion by Tamai et al. (Tamai et al., 2012). Subsequently, the study by Barouch-Bentov et al. (Barouch-Bentov et al., 2016) revealed that HCV hijacks HRS as an entry point into the ESCRT pathway to promote viral envelopment, highlighting the role of ubiquitinated NS2 residues in HRS recruitment and HCV assembly. The ubiquitination of viral proteins was found to serve as a signal for HRS binding and HCV assembly, thereby compensating for the absence of late structural domains. Additionally, HRS interacts with the viral core protein and envelope glycoproteins, facilitating their incorporation into newly formed viral particles. This interaction is crucial for the generation of infectious viral particles and the dissemination of HCV infection.

#### 3.1.4. ESCRT interacts with N protein and participates in early secretory pathway in PRRSV assembly

Porcine Reproductive and Respiratory Syndrome (PRRS) is an acute, contact-transmitted infectious disease that can cause abortion, stillbirth, weak-born piglets, mummified fetuses, and acute respiratory distress in pigs of all ages. The causative agent, Porcine Reproductive and Respiratory Syndrome Virus (PRRSV) is an enveloped, non-segmented, positive-sense RNA virus belonging to the Arteriviridae family and the genus Arterivirus (Lefkowitz et al., 2018). PRRSV infection is initiated by an interaction between the virus and host cell receptors/factors attached to the plasma membrane (Su et al., 2021). Upon internalization, PRRSV utilizes host machinery for its translation, transcription, and replication (Pasternak et al., 2006). Subsequently, the PRRSV nucleocapsid enters the endoplasmic reticulum (ER) or Golgi apparatus (GA), leading to the formation of enveloped viral particles. Finally, PRRSV viral particles are released through exocytosis, establishing a new cycle of infection. The PRRSV N protein is involved in the formation of the viral envelope by covalent and non-covalent interactions, enveloping the viral genome (Dokland, 2010). Additionally, it interacts with various host cell factors to modulate viral infection.

Among the ESCRT proteins that are crucial for PRRSV infection, TSG101 has been identified as the most upstream factor. It has been reported that TSG101 is involved in the replication of classical swine fever virus and the assembly and release of various viruses (Liu et al., 2021). As a novel host factor interacting with PRRSV N protein, TSG101 facilitates the formation of PRRSV viral particles and is involved in viral particle assembly and budding, rather than an attachment, internalization, RNA replication, and N protein translation during the infection process (Sang et al., 2012; Zhang et al., 2022). The study by Zhang et al. (2022) confirmed that inhibiting TSG101 expression or function significantly affects PRRSV replication and infectivity. Furthermore, PRRSV infection leads to the aberrant relocation and dysregulation of TSG101, affecting the balance of the ESCRT pathway and membrane trafficking within the cells.

Although significant progress has been made in elucidating the PRRSV lifecycle, a comprehensive understanding of PRRSV assembly is still lacking. The mechanisms of other identified ESCRT components involved in PRRSV infection remain unclear. Further research is needed to explore the interaction mechanisms between PRRSV and the ESCRT system to better understand the PRRSV lifecycle and viral proliferation process. Moreover, considering the importance of TSG101 in various viral infections, it holds promise as a broad-spectrum antiviral target for development.

#### 3.1.5. The role of ESCRT in the EBOV budding process

Ebola virus (EBOV) is a single-stranded negative-sense enveloped RNA virus belonging to the family Filoviridae, which can cause a severe hemorrhagic fever syndrome with a high fatality rate. The viral matrix protein VP40 is a major component of EBOV virions and is essential for filamentous particle formation (Timmins et al., 2001; Bavari et al., 2002). Oligomerization of VP40 on the plasma membrane is required for particle assembly and virus budding (Panchal et al., 2003). The late (L) budding domain of VP40 recruits hosts ESCRT (endosomal sorting complex required for transport) and ESCRT-related proteins to facilitate membrane scission, thereby mediating efficient virus-cell separation. This structural protein plays a crucial role in the late stages of virus particle assembly and release. Additionally, ESCRT-III is also critical for EBOV release. Previous studies have indicated the involvement of TSG101, a component of the ESCRT-I complex, in EBOV budding (Martin-Serrano et al., 2001). TSG101 recruitment to the plasma membrane, away from its normal functional site in endosomes, has been shown to occur through binding with EBOV VP40 (Martin-Serrano et al., 2001; Panchal et al., 2003). The interaction between the two requires specific amino acid motifs or the late (L) domain (Freed, 2002), which is responsible for the binding of TSG101 to the PTAP motif of VP40. In the final step of the vacuolar protein sorting (VPS) pathway, the ATPase activity of VPS4 generates the energy necessary for the disassembly of protein complexes, allowing for subsequent rounds of sorting. Dominant-negative VPS4 mutants lacking ATPase activity have been shown to inhibit EBOV release (Garrus et al., 2001; Licata et al., 2003). EBOV VP40 also redirects other ESCRT-I proteins from endosomes to the plasma membrane, although these interactions have not been fully characterized. Furthermore, the host ESCRT protein ALIX has been extensively implicated in the budding of retroviruses such as HIV-1 and EIAV (Martin-Serrano and Marsh, 2007; Lee et al., 2007b; Zhai et al., 2011), as well as in the egress of other RNA viruses (Irie et al., 2007). Han et al. (2015) first proposed a role for ALIX in EBOV particle budding and identified the domains of ALIX that mediate its interaction with the required VP40. Specifically, EBOV VP40 recruits host ALIX through the YPx(n)L/I motif, which can act as an alternative to the L domain in promoting virus release. These findings are of significant importance for a comprehensive understanding of the intricate virus-host interactions required for efficient EBOV egress and dissemination.

Marburg virus (MARV) is a filamentous virus capable of inducing severe hemorrhagic fever in both humans and non-human primates, with a mortality rate reaching up to 90%. Comprising seven distinct proteins, the filamentous MARV particles manifest a complex architecture. Among these constituents, the nucleoprotein (NP) encases the non-segmented negative-sense RNA genome. Coordinating with viral proteins VP35, L, VP30, and VP24, NP assembles into an elongated helical nucleocapsid (Muhlberger et al., 1998; Bharat et al., 2011). A surrounding layer of matrix protein VP40 encompasses this nucleocapsid, linking it to the viral envelope, which additionally features the incorporation of the homotrimeric glycoprotein (GP; Feldmann et al., 2001). Both matrix protein VP40 and nucleocapsid protein NP of MARV harbor specific late-domain motifs. The PSAP late-domain motif within NP plays a pivotal role in recruiting the ESCRT-I protein TSG101. Previous investigations have revealed that altering the PSAP late-domain motif within NP compromises its interaction with Tsg101, consequently impeding the liberation of MARV-specific virus-like particles (VLPs; Dolnik et al., 2010). Insights from Dolnik et al. underline the significance of the PSAP motif within NP for the efficient trafficking and envelopment of the nucleocapsid. This motif not only engages Tsg101 but also initiates the recruitment of IQGAP1, a factor involved in the regulation of the actin cytoskeleton. Notably, mutations within the PSAP motif of recombinant rMARV culminate in faulty transport processes, leading to the intracellular accumulation of the nucleocapsid and attenuating the virus’s capacity for robust intercellular dissemination (Dolnik et al., 2014).

#### 3.1.6. Significant role of ESCRT in the lifecycle of Sandfly fever viruses

The Arenaviridae family stands as a dynamically evolving and diverse collective of viruses. It encompasses an array of enveloped viruses characterized by a bipartite negative-sense RNA genome, encoding four distinct viral proteins, and marked by a non-lytic life cycle. This genome comprises two individual single-stranded RNA entities: a larger segment encoding the viral polymerase (L) and a petite zinc finger motif protein (Z), as well as a minor segment encoding both the viral nucleoprotein (NP) and the glycoprotein precursor (GPC; Southern, 1996). The Z protein, functioning as the pivotal viral matrix protein, is the prime propellant behind the budding of viral particles. In isolation, its expression can assemble VLPs (Hoenen et al., 2007). Earlier research has indicated that the proficiency of the Z protein’s budding activity correlates with the presence of late-domain motifs or aligns with the reliance on the ESCRT pathway (Perez et al., 2003; Cruz Casabona et al., 2009).

Notwithstanding the existence of diverse late-domain motifs within certain arenaviruses, the extent to which virus budding hinges on each motif appears to diverge among different viruses. For instance, the prototypic arenavirus lymphocytic choriomeningitis virus (LCMV) exclusively features a sole PPxY motif within its Z protein, a critical determinant driving the budding process (Perez et al., 2003). In contrast, the highly pathogenic Old World arenavirus Lassa virus (LASV) Z protein comprises dual PTAP and PPxY motifs. Although both motifs contribute to LASV particle release, it has been documented that the PPxY motif holds sway (Strecker et al., 2003). The research underscores the ability of diverse enveloped viruses, including members of the Arenaviridae family like LASV, to enlist ESCRT components, thereby facilitating the release of viral particles (Bieniasz, 2006; Chen and Lamb, 2008). Intriguingly, RNA interference (siRNA)-based experiments have unveiled that depletion of key factors such as HRS, TSG101, VPS22, and VPS24 substantially curbs cellular entry by LASV and LCMV (Pasqual et al., 2011). Furthermore, insights from Shtanko et al.’s investigation spotlight that ALIX/AIP1’s Bro1 domain interfaces with both NP and Z proteins. The indispensable role of ALIX is underscored in facilitating the incorporation of NP into Z-induced VLPs, as illuminated by their study (Shtanko et al., 2011).

#### 3.1.7. Regulation of assembly and budding processes of rhabdoviruses by ESCRT-associated components

Rabies virus (RABV) is a well-established zoonotic pathogen belonging to the Lyssavirus genus within the Rhabdoviridae family. Characterized by a single-stranded negative-sense RNA genome, this virus encodes a set of five essential viral proteins: nucleoprotein (N), phosphoprotein (P), matrix protein (M), glycoprotein (G), and large protein (L; Finke and Conzelmann, 2005). In the context of rhabdovirus infections, the role of the M protein is paramount—it is pivotal in the processes of budding and the formation of distinctive bullet-shaped viral particles (Mebatsion et al., 1999). Detailed proteomic analyses have unveiled the presence of ESCRT-associated factors, including CHMP4B, HSP40, Alix, TSG101, and CHMP2A, within purified RABV viral particles (Finke and Conzelmann, 2005). A recent investigation conducted by Itakura et al. has shed further light on the intricate web of interactions governing the virus’s lifecycle. Specifically, their research has illuminated the pivotal involvement of the ESCRT-I protein, TSG101, in the later stages of this lifecycle. TSG101 has emerged as a key binding partner for the RABV M protein. The concerted effort between the L domain of TSG101 and the L structure domain of RABV M is instrumental in orchestrating the efficient assembly, budding, and distinctive bullet-shaped morphology that characterize RABV (Itakura et al., 2023).

Infectious hematopoietic necrosis virus (IHNV), much like RABV, resides within the Rhabdoviridae family and boasts a negative-sense single-stranded RNA genome. This genomic structure encodes a cluster of six distinctive proteins: nucleoprotein (N), polymerase-associated phosphoprotein (P), matrix protein (M), unique glycoprotein (G), large RNA-dependent RNA polymerase (L), and a concise intergenic region nestled between the G and L genes (Morzunov et al., 1995; Assenberg et al., 2010; Kim and Kim, 2011). Throughout viral replication, the M protein kickstarts the intricate choreography of viral particle assembly and budding (Dancho et al., 2009). The G protein’s budding domain, in turn, orchestrates either the curvature of membranes at the budding site or the strategic aggregation of nucleocapsids onto the plasma membrane. This dual approach seamlessly promotes the liberation of viruses (Robison and Whitt, 2000). Pioneering the exploration of IHNV budding, Chen et al. (2019) stumbled upon the influential role of VPS4. A remarkable impact on IHNV budding emerged in response to siRNA-induced VPS4B attenuation. In stark contrast, when VPS4A was downregulated, IHNV budding seemed relatively unaltered. This body of evidence underscores the pivotal involvement of VPS4B in IHNV budding while suggesting a less pivotal role for VPS4A in IHNV’s emancipation. In parallel, Chen et al. unveiled a network of interactions between IHNV’s M, G, and L proteins and Nedd4, Tsg101, and Alix. Collectively, these discoveries underscore the paramount importance of interactions between virus proteins bearing the L domain and their corresponding E-class proteins in IHNV budding. Furthermore, the host factors at play exert their influential touch upon the intricate ballet of IHNV’s budding journey.

#### 3.1.8. Involvement of the ESCRT pathway in CSFV entry, replication, and virion formation

Classical swine fever (CSF), caused by classical swine fever virus (CSFV), is a highly contagious disease of swine with high morbidity and mortality (Moennig, 2015). CSFV is an enveloped, positive-stranded RNA virus that belongs to the genus Pestivirus within the family Flaviviridae (Meyers et al., 1996). The viral genome is a single-stranded +RNA of approximately 12.3 kb with a structure of a single open reading frame (ORF) surrounded by two untranslated regions (UTRs). The ORF encodes a polyprotein that is cleaved into four structural (capsid protein C, envelope glycoproteins Erns, E1, and E2) and eight nonstructural proteins (Npro, p7, NS2, NS3, NS4A, NS4B, NS5A, and NS5B; Zhou, 2019; Fan et al., 2021). After CSFV enters the host cells, a series of reactions occur with intracellular components that regulate different stages of the viral life cycle, including entry, trafficking, replication, assembly, and release (Ning et al., 2016; Zheng et al., 2020). ESCRT-I complex Tsg101 protein participates in clathrin-mediated endocytosis of CSFV and is also involved in CSFV trafficking. Tsg101 assists the virus in entering the host cell through the late endosome (Rab7 and Rab9) and finally reaching the lysosome (Lamp-1). Interestingly, Tsg101 is also involved in the viral replication process by interacting with nonstructural proteins 4B and 5B of CSFV (Liu et al., 2021).

Liu et al. investigated the Tsg101, VPS25, CHMP4B, and CHMP7 proteins that play essential roles in viral trafficking and Clathrin-mediated viral entry. CHMP4B and CHMP7 interact with Rab5 to transport CSFV virions from early endosomes to late endosomes. They first demonstrate that HRS (ESCRT-0), VPS28 (ESCRT-I), VPS25 (ESCRT-II), and adaptor protein ALIX play important roles in the formation of virus replication complexes (VRC) together with CHMP2B/4B/7 (ESCRT-III), and VPS4A. Tsg101 and CHMP4B are associated with LAMP-1 in the lysosomes, probably leading to uncoating and release of nucleic acid for genome replication in the endoplasmic reticulum area (Liu et al., 2022).

### 3.2. ESCRT and enveloped DNA viruses

In addition to enveloped RNA viruses, the ESCRT complex is also involved in the assembly and release processes of enveloped DNA viruses. For instance, Hepatitis B virus (HBV) and Herpes simplex virus type 1 (HSV-1).

#### 3.2.1. Involvement of ESCRT in the HBV infection process

HBV is the major pathogen responsible for human liver disease. It is an enveloped, DNA-containing pararetrovirus that replicates primarily through reverse transcription of its pregenomic RNA (pgRNA; Stieler and Prange, 2014). Despite the success of global HBV vaccination programs, a definitive cure for HBV infection has yet to be found (Ringelhan et al., 2015; Thomas et al., 2015). Persistent HBV infection can lead to the development of liver cirrhosis, liver damage, and hepatocellular carcinoma (Ganem and Prince, 2004). When the nucleocapsid of HBV reaches genomic maturation in the cytoplasm, two distinct outcomes can occur: one involves encapsidation of the viral particles and their release through the ESCRT system, while the other involves the return of the viral RC DNA genome to the cell nucleus for further amplification of covalently closed circular DNA (Tuttleman et al., 1986).

During HBV infection, all components of ESCRT-0 are essential for HBV replication. The core antigen of HBV (HBcAg) interacts with ESCRT-0, facilitating the assembly and release of HBV particles. Specifically, the HBcAg protein forms a complex with the HRS subunit of ESCRT-0, which promotes the intracellular aggregation and assembly of HBV core proteins, ultimately leading to the release of HBV particles. Additionally, the surface antigen of HBV (HBsAg) can interact with the ESCRT complex, facilitating the release of HBV particles. Studies by Chou et al. (Chou et al., 2015) have shown that both downregulation and overexpression of HRS result in the inhibition of HBV RNA transcription, DNA replication, and virus particle production, partly through promoting naked capsid secretion.

Due to the absence of the P (S/T)AP-like late-domain motif in HBV structural proteins, which is known to interact with TSG101, it was previously believed that TSG101 was dispensable for HBV export. However, recent research findings have demonstrated that the knockdown of TSG101 reduces extracellular HBV DNA levels and results in the accumulation of viral capsids within cells. This supports the essential role of TSG101 in the process of HBV extracellular capsid release (Zheng et al., 2023). Although the absence of ESCRT-II does not impair the capsid assembly reaction, it selectively impairs the formation and/or stability of nuclear capsids. In cells depleted of ESCRT-II, the levels of enveloped pgRNA are significantly reduced, indicating that ESCRT-II guides steps in capsid formation that accompany replicative capacities, such as facilitating RNA transport and capsid envelopment. Stieler et al. performed knockdown experiments targeting ESCRT-II components EAP20, EAP30, and EAP45, and found that depletion of ESCRT-II components dramatically reduced virus budding. Therefore, depletion of ESCRT-II components EAP20, EAP30, and EAP45 not only inhibits the production and/or release of enveloped virus particles but also impairs the formation of intracellular nuclear capsids. HBV budding on cellular membranes also requires the involvement of ESCRT-III and the VPS4 complex in membrane scission and separation (Stieler and Prange, 2014).

#### 3.2.2. The link between HSV-1 and the ESCRT system

HSV-1 is a structurally complex enveloped double-stranded DNA virus belonging to the Herpesviridae family. It is a human pathogen that can cause diseases such as oral herpes, keratitis, and encephalitis. Its capsid can package together with the DNA genome in the cell nucleus (Dai and Zhou, 2018). The viral capsid undergoes primary packaging at the inner nuclear membrane (Bigalke and Heldwein, 2017) and then undergoes final secondary packaging within the lumen of cellular compartments in the cytoplasm (Hollinshead et al., 2012). During this process, it utilizes its viral-encoded proteins and the cellular ESCRT mechanism to drive its envelope assembly. ESCRT-III and VPS4 are essential for HSV-1 to accomplish cytoplasmic secondary packaging and play important roles in the wrapping process of HSV-1 at the inner nuclear membrane (Crump et al., 2007; Pawliczek and Crump, 2009).

Generally, viruses recruit one or more early ESCRT components to access ESCRT-III, with the most common being ALIX and the ESCRT-I complex (Votteler and Sundquist, 2013). However, unlike many other members of the enveloped virus family, HSV-1 appears to not require the ESCRT-I subunit TSG101 or the protein ALIX containing the Bro1 domain for recruitment and activation of ESCRT-III. Both of them are dispensable for HSV-1 envelopment even when simultaneously depleted (Pawliczek and Crump, 2009). Its structural components may directly interact with ESCRT-II to control ESCRT-III assembly. Following this, Jenna Barnes and colleagues proposed a hypothesis that due to the lack of ALIX and ESCRT-I involvement, the most likely remaining cellular proteins used by HSV-1 to assemble the ESCRT-III machinery are the ESCRT-II complex and Bro1 family members other than ALIX. Knockdown experiments targeting the critical EAP25/VPS1 subunit of ESCRT-II and the remaining known ESCRT-related Bro1 domain proteins, HD-PTP and BROX, revealed no impact on HSV-1 replication (Barnes and Wilson, 2020).

Considering the aforementioned findings, it is reasonable to hypothesize that ESCRT-III may be directly recruited by proteins present in the capsid or envelope of HSV-1. HSV-1 appears to redundantly utilize multiple cellular ESCRT components for ESCRT-III recruitment, or the virus may completely bypass the requirement for Bro1 domain proteins, ESCRT-I, and ESCRT-II. In the latter scenario, the structural proteins within HSV-1 particles, whether internal or external to the capsid, might have evolved to directly recruit and trigger the assembly of ESCRT-III subunits at sites of envelope constriction and scission (Crump, 2018; Barnes and Wilson, 2019). The interaction between HSV-1 and the ESCRT system may involve the participation of other components, and further investigation is needed to elucidate the specific mechanisms and their implications.

#### 3.2.3. The roles of cellular ESCRT proteins in efficient entry and egress of budded virions of *Autographa californica* multiple nucleopolyhedrovirus

Autographa California Multiple Nuclear Polyhedrovirus (AcMNPV) is the most intensively studied baculovirus and is the type species of the virus family Baculoviridae. Baculoviruses are enveloped, insect-specific double-stranded DNA (dsDNA) viruses that replicate in the nuclei of infected cells. During the infection cycle, baculoviruses produce two phenotypes of enveloped virions: occlusion-derived virions (ODV) and budded virions (BV). The ESCRT pathway is conserved in sequenced insect genomes and the expression levels of certain components of ESCRT-I, -II, -III, Vps4, and Alix were significantly upregulated upon AcMNPV infection (Chen et al., 2014; Li and Blissard, 2015).

Prior studies revealed that efficient entry and egress of AcMNPV BV are dependent on functional Vps4 (Li and Blissard, 2012). Since Vps4 is required for recycling ESCRT III and represents a terminal step in the ESCRT pathway, this suggests that other components of the ESCRT pathway may be specifically involved in entry and egress. However, In the current study, Yue et al. found that functional ESCRT-I and ESCRT-III complexes were required for efficient entry and transport of AcMNPV budded virions early in infection. The components of ESCRT-III (but not ESCRT-I) are also necessary for the efficient nuclear egress of progeny nucleocapsids. In addition, Yue et al. also found that several baculovirus cores or conserved proteins (Ac11, Ac76, Ac78, GP41, Ac93, Ac103, Ac142, and Ac146) interact with Vps4 and components of ESCRT-III. These viral proteins may form an “egress complex” that is involved in recruiting ESCRT-III components to a virus-egress domain on the nuclear membrane (Yue et al., 2018).

## 4. Discussion

ESCRT plays a crucial role in various cellular processes, including the budding of enveloped viruses, regulation of innate immune responses, and degradation of damaged or unwanted cellular components. Although significant progress has been made in understanding the molecular mechanisms of ESCRT-mediated virus budding, much remains to be explored. For instance, the molecular mechanisms underlying the regulation of ESCRT components’ expression and activity require further investigation. Moreover, the precise roles of ESCRT in virus replication and pathogenesis need to be elucidated. An enhanced understanding of how the ESCRT system is hijacked by viruses can greatly contribute to our knowledge of normal cellular physiological functions.

In the future, research on ESCRT function in virus-host interactions will provide new insights into the development of antiviral therapies. A better understanding of the ESCRT complex’s role in innate immune response regulation will lead to the development of novel approaches to treating viral infections. Moreover, the identification and characterization of novel ESCRT-interacting viral proteins and their interaction mechanisms will expand our knowledge of virus-host interactions. Finally, the development of new technologies, such as single-particle cryo-electron microscopy, will allow us to determine the three-dimensional structure of ESCRT and provide a more detailed understanding of its molecular mechanisms. Overall, further research on ESCRT and its interactions with viruses will help to develop new strategies to combat viral infections.

## Author contributions

CW: Writing – original draft. YC: Writing – review & editing. SH: Writing – review & editing. XL: Writing – review & editing.
